# Supplementary material for: Adipose tissue in health and disease through the lens of its building blocks
Source: Sci Rep. 2020 Jun 26;10:10433. doi: 10.1038/s41598-020-67177-1 (PMC7319996; doi:10.1038/s41598-020-67177-1)
Supplement: Supplementary file 6 — Supplementary Figures [file 41598_2020_67177_MOESM6_ESM.pdf]

Supplementary Figures

Adipose tissue in health and disease through the lens of its building blocks

Authors: Michael Lenz (ML)[1,2,3,‡] , Ilja C.W. Arts (IA)[1,4], Ralf L.M. Peeters (RP)[1,5], Theo M. de Kok (TK)[1,6] and Gökhan Ertaylan (GE)[1,7,‡]

Affiliations:

[1]Maastricht Centre for Systems Biology (MaCSBio), Maastricht University, the Netherlands

[2]Institute of Organismic and Molecular Evolution, Johannes Gutenberg University Mainz, Mainz, Germany

[3]Preventive Cardiology and Preventive Medicine – Center for Cardiology, University Medical Center of the Johannes Gutenberg-University Mainz, Mainz, Germany

[4]Dept. of Epidemiology, CARIM School of Cardiovascular Diseases, Maastricht University, the Netherlands

[5]Dept. of Data Science & Knowledge Engineering, Maastricht University, the Netherlands

[6]Dept. of Toxicogenomics, GROW School of Oncology and Developmental Biology, Maastricht University, Maastricht, The Netherlands

[7]Health, Flemish Institute for Technological Research (VITO), Mol, Belgium

[‡]Corresponding author

Email(s): ML: mlenz@uni-mainz.de;

IA: ilja.arts@maastrichtuniversity.nl;

RP: ralf.peeters@maastrichtuniversity.nl;

TK: t.dekok@maastrichtuniversity.nl;

GE: gokhan.ertaylan@vito.be

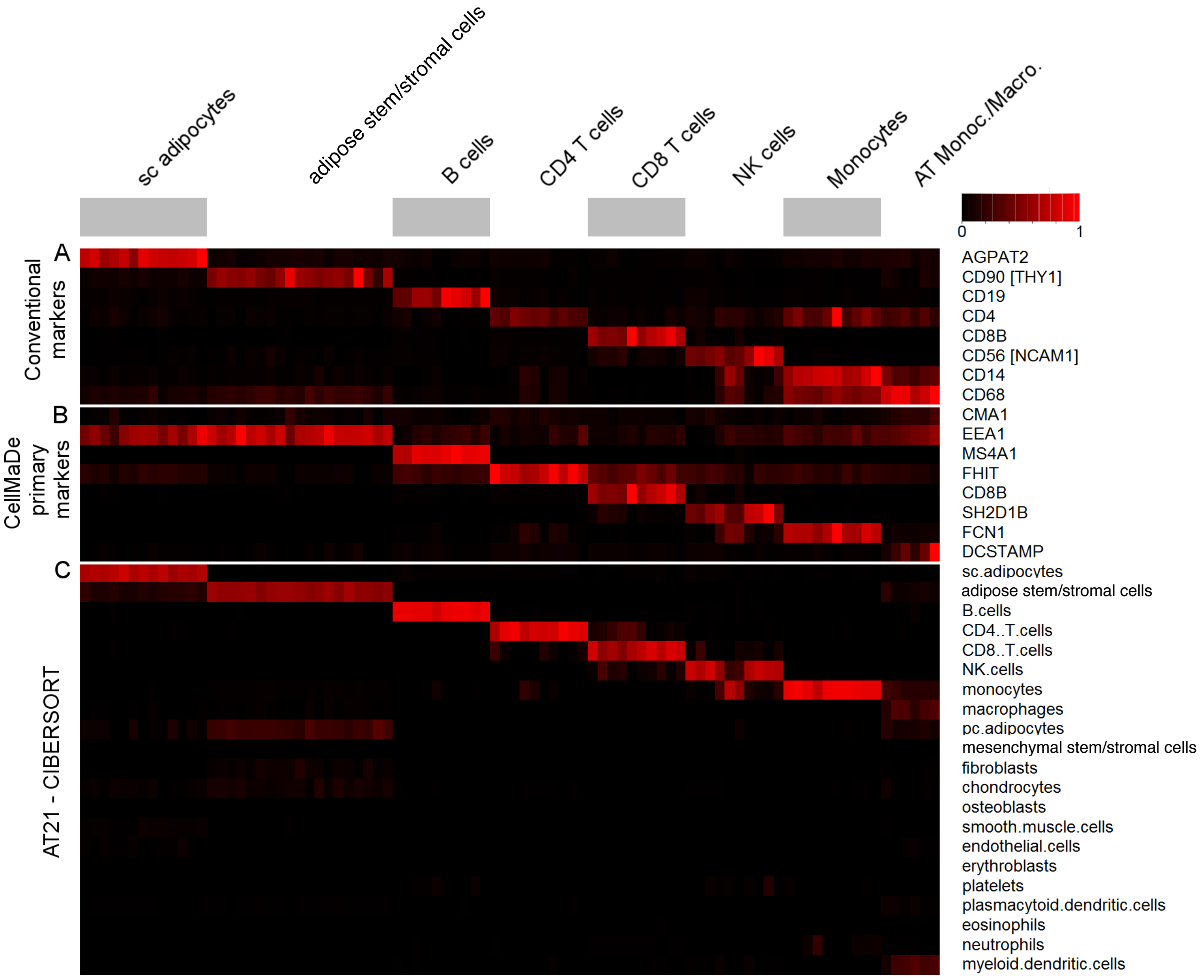

Supplementary Figure S1 : Ex-vivo validation of the TissueDecoder framework with an independent dataset. A) Normalized expression of selected conventional markers from literature; B) normalized expression of CellMaDe predicted primary markers; and C) cell type composition prediction from CIBERSORT with the AT21 signature matrix. The common x-axis above the figure denotes the reported cell type of the samples from the validation dataset.

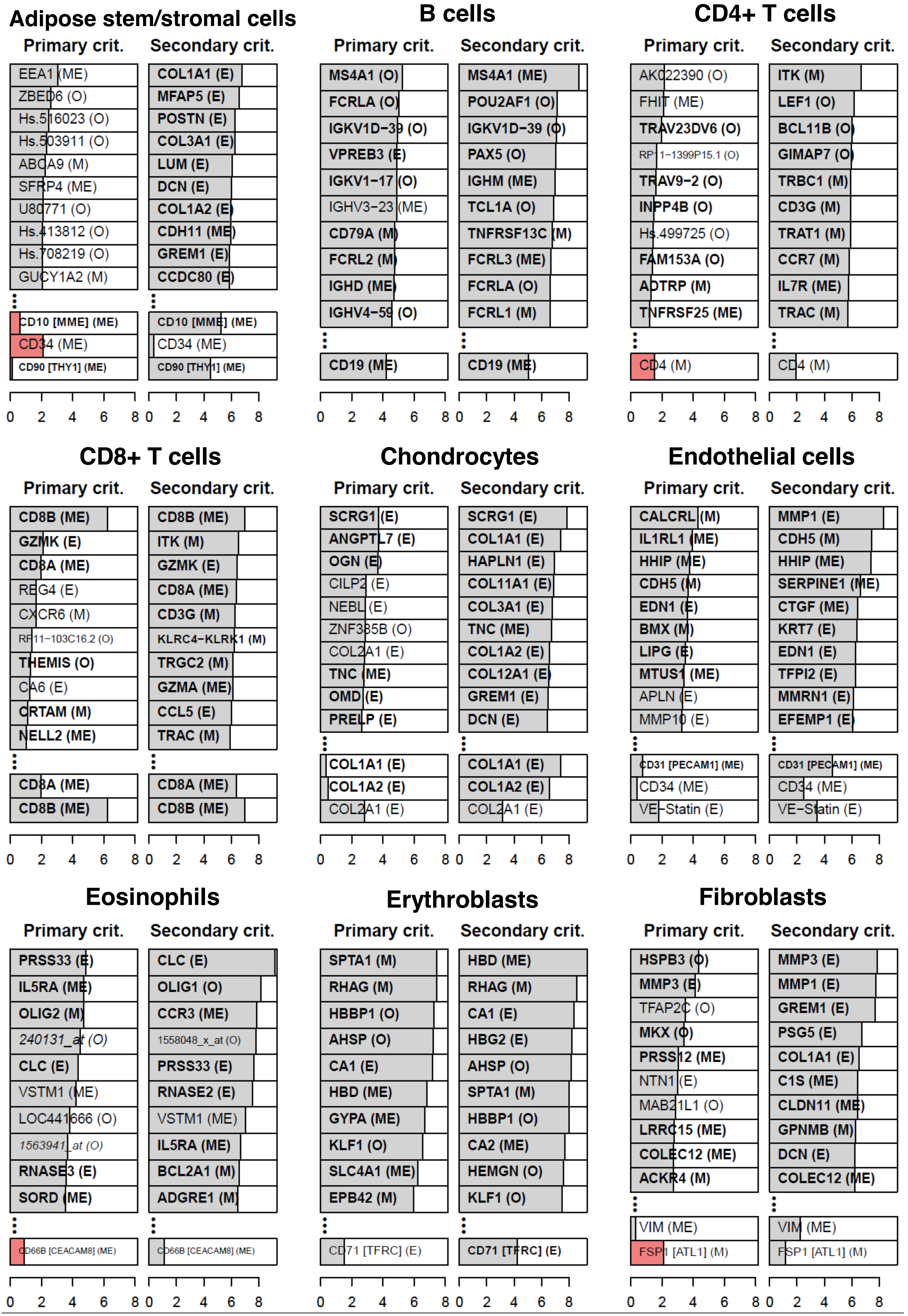

Adipose stem/stromal cells
Primary crit.
EEA1 (ME)
ZBED6 (O)
Hs.516023 (O)
Hs.503911 (O)
ABCA9 (M)
SFRP4 (ME)
U80771 (O)
Hs.413812 (O)
Hs.708219 (O)
GUCY1A2 (M)
CD10 [MME] (ME)
CD34 (ME)
CD90 [THY1] (ME)
Secondary crit.
COL1A1 (E)
MFAP5 (E)
POSTN (E)
COL3A1 (E)
LUM (E)
DCN (E)
COL1A2 (E)
CDH11 (ME)
GREM1 (E)
CCDC80 (E)
CD10 [MME] (ME)
CD34 (ME)
CD90 [THY1] (ME)
B cells
Primary crit.
MS4A1 (O)
FCRLA (O)
IGKV1D−39 (O)
VPREB3 (E)
IGKV1−17 (O)
IGHV3−23 (ME)
CD79A (M)
FCRL2 (M)
IGHD (ME)
IGHV4−59 (O)
CD19 (ME)
Secondary crit.
MS4A1 (ME)
POU2AF1 (O)
IGKV1D−39 (O)
PAX5 (O)
IGHM (ME)
TCL1A (O)
TNFRSF13C (M)
FCRL3 (ME)
FCRLA (O)
FCRL1 (M)
CD19 (ME)
CD4+ T cells
Primary crit.
AK022390 (O)
FHIT (ME)
TRAV23DV6 (O)
RP11−1399P15.1 (O)
TRAV9−2 (O)
INPP4B (O)
Hs.499725 (O)
FAM153A (O)
ADTRP (M)
TNFRSF25 (ME)
CD4 (M)
Secondary crit.
ITK (M)
LEF1 (O)
BCL11B (O)
GIMAP7 (O)
TRBC1 (M)
CD3G (M)
TRAT1 (M)
CCR7 (M)
IL7R (ME)
TRAC (M)
CD4 (M)
CD8+ T cells
Primary crit.
CD8B (ME)
GZMK (E)
CD8A (ME)
REG4 (E)
CXCR6 (M)
RP11−103C16.2 (O)
THEMIS (O)
CA6 (E)
CRTAM (M)
NELL2 (ME)
CD8A (ME)
CD8B (ME)
Secondary crit.
CD8B (ME)
ITK (M)
GZMK (E)
CD8A (ME)
CD3G (M)
KLRC4−KLRK1 (M)
TRGC2 (M)
GZMA (ME)
CCL5 (E)
TRAC (M)
CD8A (ME)
CD8B (ME)
Chondrocytes
Primary crit.
SCRG1 (E)
ANGPTL7 (E)
OGN (E)
CILP2 (E)
NEBL (E)
ZNF385B (O)
COL2A1 (E)
TNC (ME)
OMD (E)
PRELP (E)
COL1A1 (E)
COL1A2 (E)
COL2A1 (E)
Secondary crit.
SCRG1 (E)
COL1A1 (E)
HAPLN1 (E)
COL11A1 (E)
COL3A1 (E)
TNC (ME)
COL1A2 (E)
COL12A1 (E)
GREM1 (E)
DCN (E)
COL1A1 (E)
COL1A2 (E)
COL2A1 (E)
Endothelial cells
Primary crit.
CALCRL (M)
IL1RL1 (ME)
HHIP (ME)
CDH5 (M)
EDN1 (E)
BMX (M)
LIPG (E)
MTUS1 (ME)
APLN (E)
MMP10 (E)
CD31 [PECAM1] (ME)
CD34 (ME)
VE−Statin (E)
Secondary crit.
MMP1 (E)
CDH5 (M)
HHIP (ME)
SERPINE1 (ME)
CTGF (ME)
KRT7 (E)
EDN1 (E)
TFPI2 (E)
MMRN1 (E)
EFEMP1 (E)
CD31 [PECAM1] (ME)
CD34 (ME)
VE−Statin (E)
Eosinophils
Primary crit.
PRSS33 (E)
IL5RA (ME)
OLIG2 (M)
240131_at (O)
CLC (E)
VSTM1 (ME)
LOC441666 (O)
1563941_at (O)
RNASE3 (E)
SORD (ME)
CD66B [CEACAM8] (ME)
Secondary crit.
CLC (E)
OLIG1 (O)
CCR3 (ME)
1558048_x_at (O)
PRSS33 (E)
RNASE2 (E)
VSTM1 (ME)
IL5RA (ME)
BCL2A1 (M)
ADGRE1 (M)
CD66B [CEACAM8] (ME)
Erythroblasts
Primary crit.
SPTA1 (M)
RHAG (M)
HBBP1 (O)
AHSP (O)
CA1 (E)
HBD (ME)
GYPA (ME)
KLF1 (O)
SLC4A1 (ME)
EPB42 (M)
CD71 [TFRC] (E)
Secondary crit.
HBD (ME)
RHAG (M)
CA1 (E)
HBG2 (E)
AHSP (O)
SPTA1 (M)
HBBP1 (O)
CA2 (ME)
HEMGN (O)
KLF1 (O)
CD71 [TFRC] (E)
Fibroblasts
Primary crit.
HSPB3 (O)
MMP3 (E)
TFAP2C (O)
MKX (O)
PRSS12 (ME)
NTN1 (E)
MAB21L1 (O)
LRRC15 (ME)
COLEC12 (ME)
ACKR4 (M)
VIM (ME)
FSP1 [ATL1] (M)
Secondary crit.
MMP3 (E)
MMP1 (E)
GREM1 (E)
PSG5 (E)
COL1A1 (E)
C1S (ME)
CLDN11 (ME)
GPNMB (M)
DCN (E)
COLEC12 (ME)
VIM (ME)
FSP1 [ATL1] (M)
0 2 4 6 8

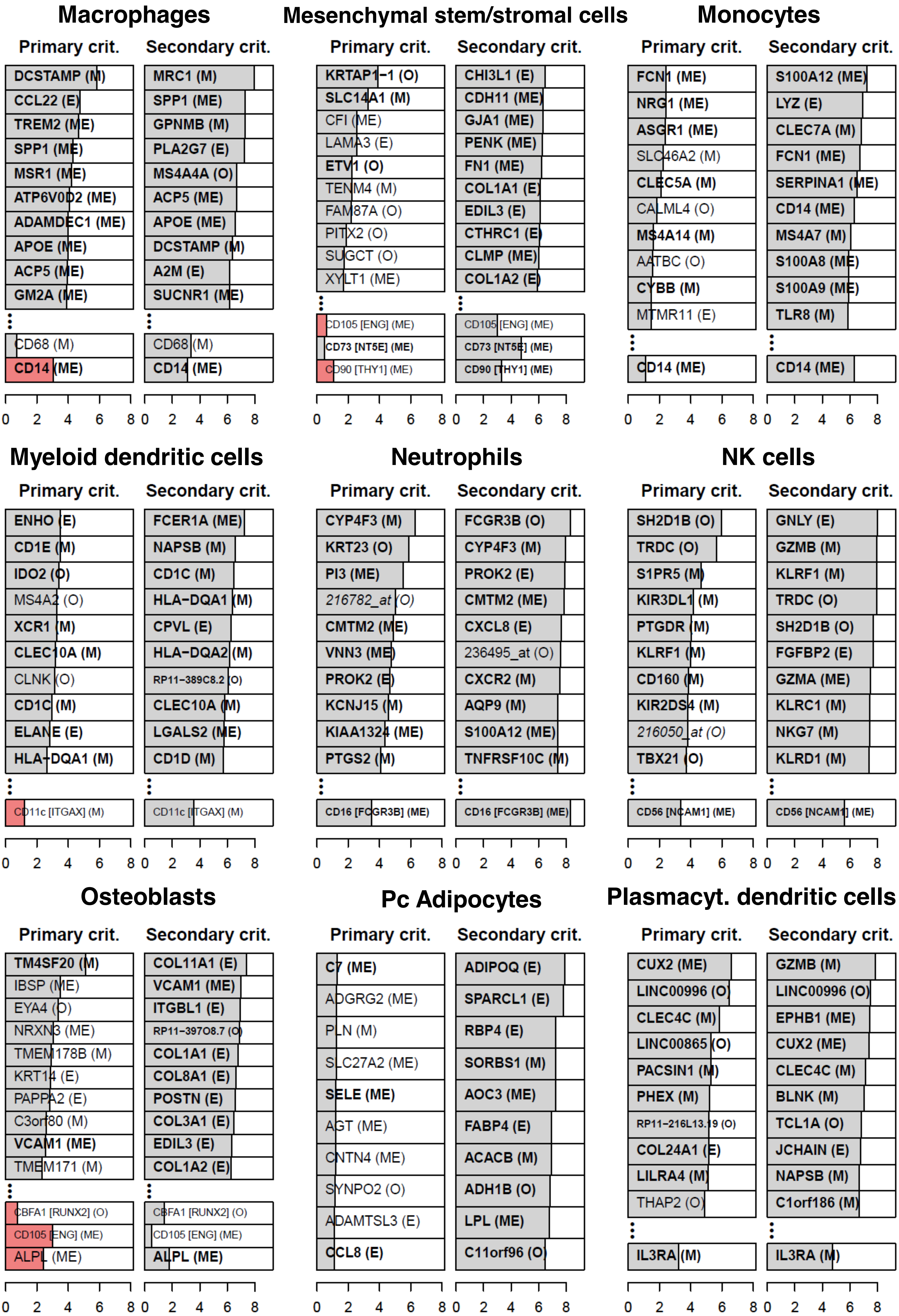

Macrophages
Primary crit.
DCSTAMP (M)
CCL22 (E)
TREM2 (ME)
SPP1 (ME)
MSR1 (ME)
ATP6V0D2 (ME)
ADAMDEC1 (ME)
APOE (ME)
ACP5 (ME)
GM2A (ME)
CD68 (M)
CD14 (ME)
Secondary crit.
MRC1 (M)
SPP1 (ME)
GPNMB (M)
PLA2G7 (E)
MS4A4A (O)
ACP5 (ME)
APOE (ME)
DCSTAMP (M)
A2M (E)
SUCNR1 (ME)
CD68 (M)
CD14 (ME)
Mesenchymal stem/stromal cells
Primary crit.
KRTAP1−1 (O)
SLC14A1 (M)
CFI (ME)
LAMA3 (E)
ETV1 (O)
TENM4 (M)
FAM87A (O)
PITX2 (O)
SUGCT (O)
XYLT1 (ME)
CD105 [ENG] (ME)
CD73 [NT5E] (ME)
CD90 [THY1] (ME)
Secondary crit.
CHI3L1 (E)
CDH11 (ME)
GJA1 (ME)
PENK (ME)
FN1 (ME)
COL1A1 (E)
EDIL3 (E)
CTHRC1 (E)
CLMP (ME)
COL1A2 (E)
CD105 [ENG] (ME)
CD73 [NT5E] (ME)
CD90 [THY1] (ME)
Monocytes
Primary crit.
FCN1 (ME)
NRG1 (ME)
ASGR1 (ME)
SLC46A2 (M)
CLEC5A (M)
CALML4 (O)
MS4A14 (M)
AATBC (O)
CYBB (M)
MTMR11 (E)
CD14 (ME)
Secondary crit.
S100A12 (ME)
LYZ (E)
CLEC7A (M)
FCN1 (ME)
SERPINA1 (ME)
CD14 (ME)
MS4A7 (M)
S100A8 (ME)
S100A9 (ME)
TLR8 (M)
CD14 (ME)
Myeloid dendritic cells
Primary crit.
ENHO (E)
CD1E (M)
IDO2 (O)
MS4A2 (O)
XCR1 (M)
CLEC10A (M)
CLNK (O)
CD1C (M)
ELANE (E)
HLA−DQA1 (M)
CD11c [ITGAX] (M)
Secondary crit.
FCER1A (ME)
NAPSB (M)
CD1C (M)
HLA−DQA1 (M)
CPVL (E)
HLA−DQA2 (M)
RP11−389C8.2 (O)
CLEC10A (M)
LGALS2 (ME)
CD1D (M)
CD11c [ITGAX] (M)
Neutrophils
Primary crit.
CYP4F3 (M)
KRT23 (O)
PI3 (ME)
216782_at (O)
CMTM2 (ME)
VNN3 (ME)
PROK2 (E)
KCNJ15 (M)
KIAA1324 (ME)
PTGS2 (M)
CD16 [FCGR3B] (ME)
Secondary crit.
FCGR3B (O)
CYP4F3 (M)
PROK2 (E)
CMTM2 (ME)
CXCL8 (E)
236495_at (O)
CXCR2 (M)
AQP9 (M)
S100A12 (ME)
TNFRSF10C (M)
CD16 [FCGR3B] (ME)
NK cells
Primary crit.
SH2D1B (O)
TRDC (O)
S1PR5 (M)
KIR3DL1 (M)
PTGDR (M)
KLRF1 (M)
CD160 (M)
KIR2DS4 (M)
216050_at (O)
TBX21 (O)
CD56 [NCAM1] (ME)
Secondary crit.
GNLY (E)
GZMB (M)
KLRF1 (M)
TRDC (O)
SH2D1B (O)
FGFBP2 (E)
GZMA (ME)
KLRC1 (M)
NKG7 (M)
KLRD1 (M)
CD56 [NCAM1] (ME)
Osteoblasts
Primary crit.
TM4SF20 (M)
IBSP (ME)
EYA4 (O)
NRXN3 (ME)
TMEM178B (M)
KRT14 (E)
PAPPA2 (E)
C3orf80 (M)
VCAM1 (ME)
TMEM171 (M)
CBFA1 [RUNX2] (O)
CD105 [ENG] (ME)
ALPL (ME)
Secondary crit.
COL11A1 (E)
VCAM1 (ME)
ITGBL1 (E)
RP11−397O8.7 (O)
COL1A1 (E)
COL8A1 (E)
POSTN (E)
COL3A1 (E)
EDIL3 (E)
COL1A2 (E)
CBFA1 [RUNX2] (O)
CD105 [ENG] (ME)
ALPL (ME)
Pc Adipocytes
Primary crit.
C7 (ME)
ADGRG2 (ME)
PLN (M)
SLC27A2 (ME)
SELE (ME)
AGT (ME)
CNTN4 (ME)
SYNPO2 (O)
ADAMTSL3 (E)
CCL8 (E)
Secondary crit.
ADIPOQ (E)
SPARCL1 (E)
RBP4 (E)
SORBS1 (M)
AOC3 (ME)
FABP4 (E)
ACACB (M)
ADH1B (O)
LPL (ME)
C11orf96 (O)
Plasmacyt. dendritic cells
Primary crit.
CUX2 (ME)
LINC00996 (O)
CLEC4C (M)
LINC00865 (O)
PACSIN1 (M)
PHEX (M)
RP11−216L13.19 (O)
COL24A1 (E)
LILRA4 (M)
THAP2 (O)
IL3RA (M)
Secondary crit.
GZMB (M)
LINC00996 (O)
EPHB1 (ME)
CUX2 (ME)
CLEC4C (M)
BLNK (M)
TCL1A (O)
JCHAIN (E)
NAPSB (M)
C1orf186 (M)
IL3RA (M)
0 2 4 6 8

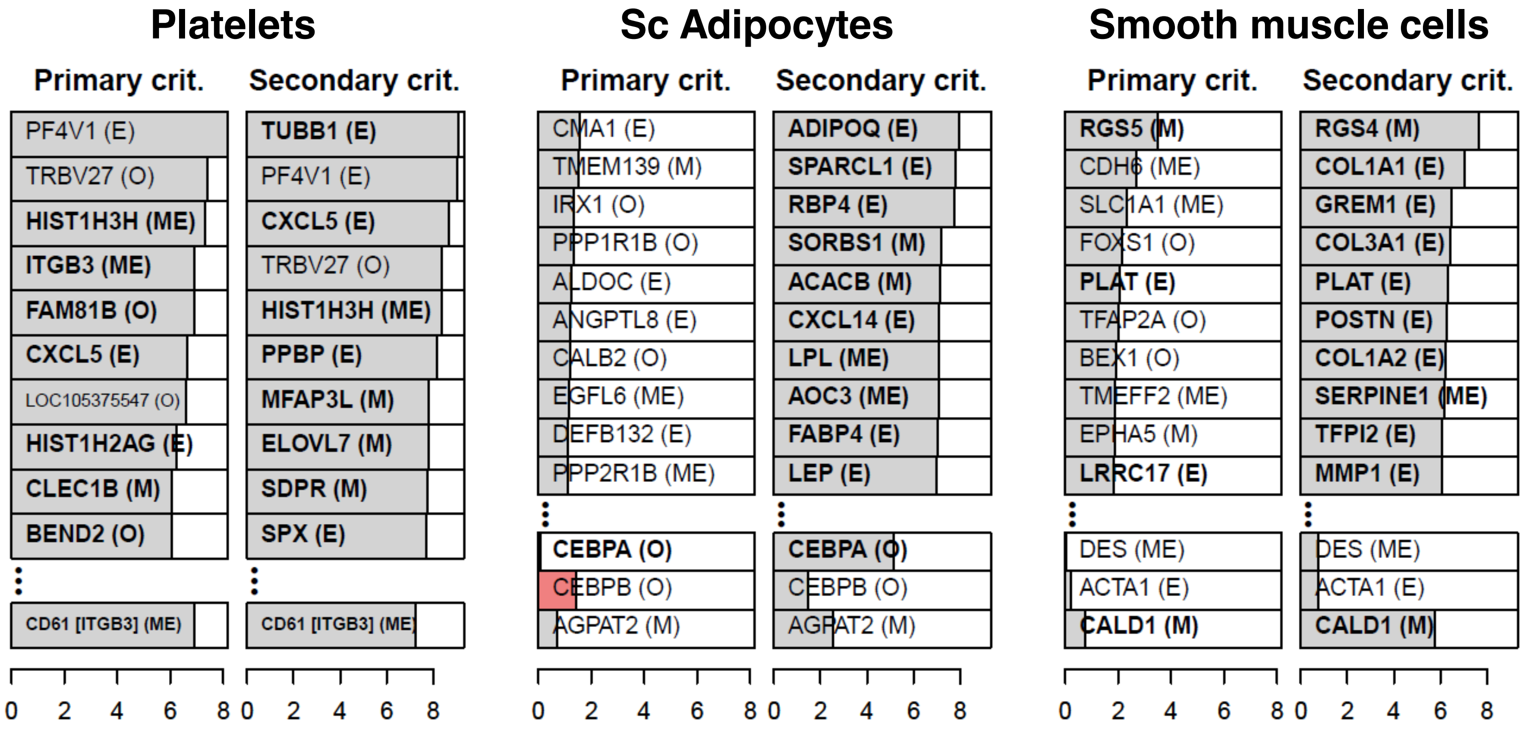

Supplementary Figure S2: Primary and secondary markers for all 21 cell types. Bar plots show the top ten cell type markers identified by the primary criterion and the secondary criterion of CellMaDe. Below each column conventional markers are demonstrated with their associated score. **Bold** font indicates the presence of the gene in the AT21 signature matrix and red colored bars indicate the value of the (primary/secondary) criterion was negative. Letters in brackets (M: membrane, E: extracellular, ME: both membrane and extracellular, or O: other) specify the gene ontology cellular location of the corresponding protein.

Supplementary Figure S3. Tissue expressions of the most significant Primary Marker per cell type identified by CellMaDe analyzed by Genevestigator from 394 human anatomically annotated tissue expression profiles.
The genes are predicted to be expressed in A) Subcutaneous Adipocytes, B)Pericardial Adipocytes, C) A. Stromal/ Stem Cells, D) Mesenchymal stromal/stem Cells, E) Fibroblasts, F) Chondrocytes, G) Osteoblasts, H) Smooth Muscle Cells, I) Endothelial Cells, J) Erytrhoblasts, K) Platelets, L) Natural Killer Cells, M) CD4+ T cells, N) CD8+ T cells, O) B Cells, P) Plasmacytoid Dendritic Cells, Q) Eosinophils, R) Neutrophils, S) Myeloid Dendritic Cells, U) Monocyes, V) Macrophages.

*The data is accessed on 12/08/2017.

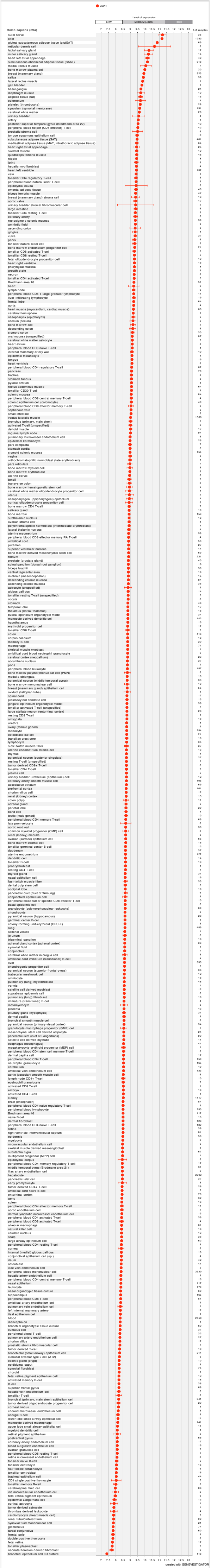

A) Subcutaneous Adipocytes

B) Pericardial Adipocytes

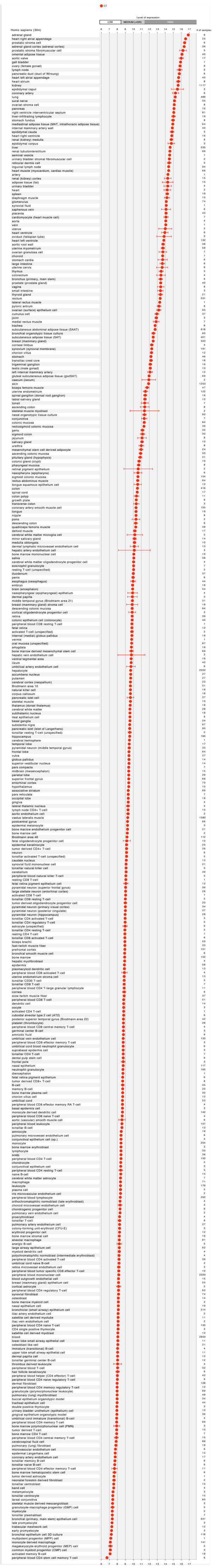

C) Adipose Stem/Stromal Cells

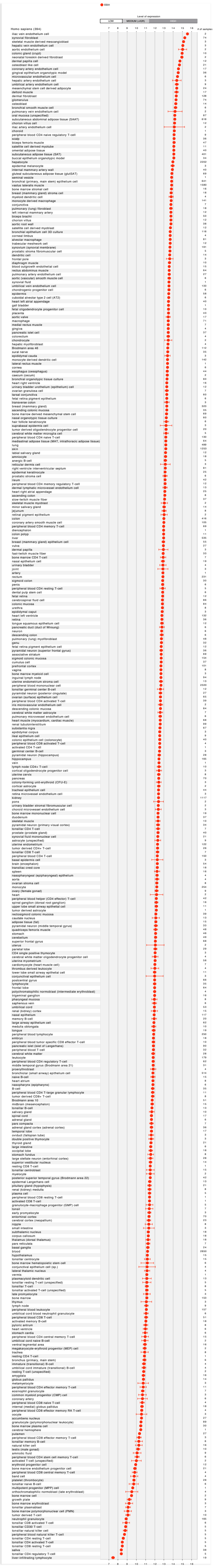

C) Mesenchymal Stem/Stromal Cells

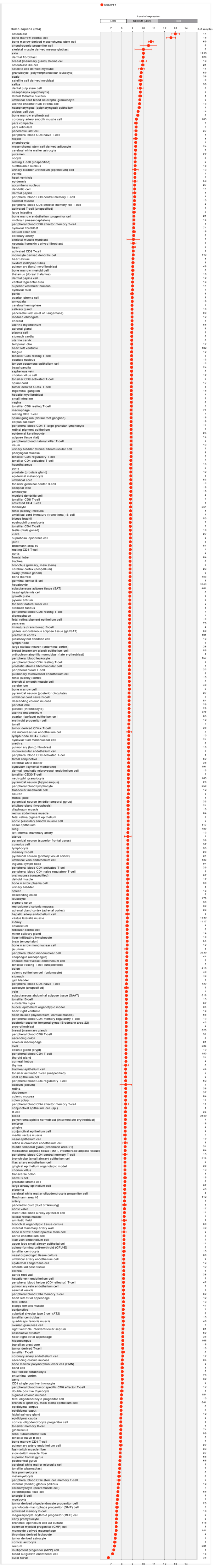

E) Fibroblasts

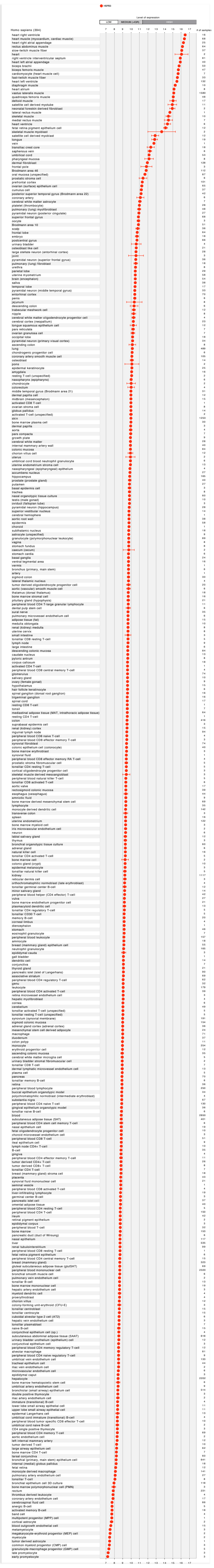

F) Chondrocytes

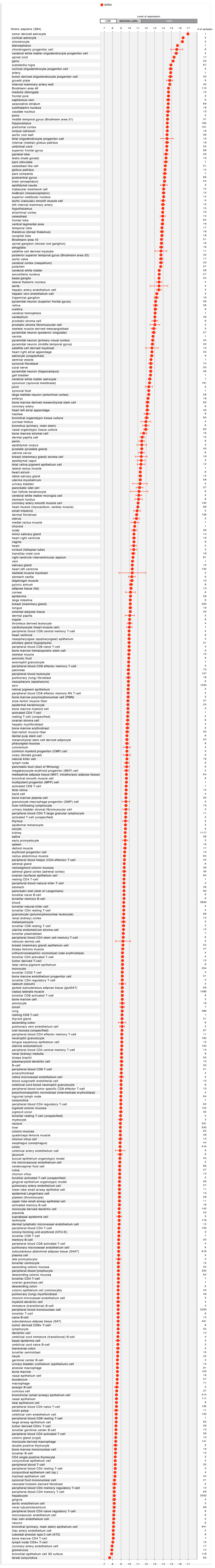

G) Osteoblasts

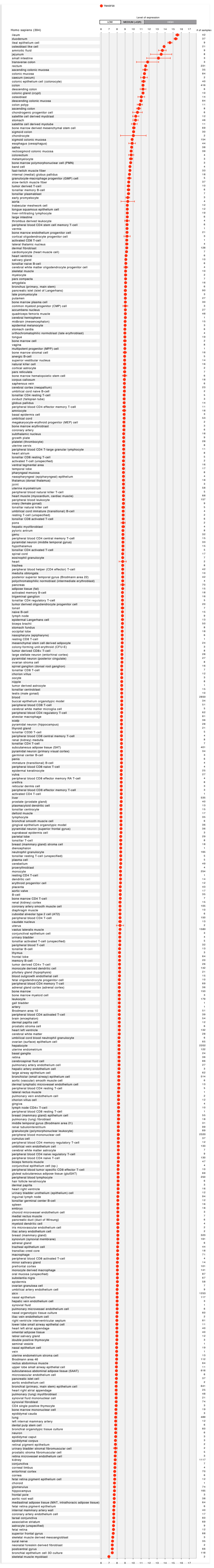

H) Smooth Muscle Cells

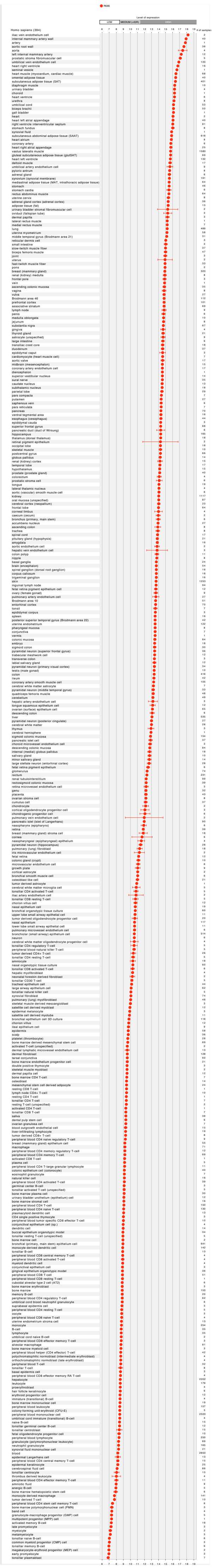

I) Endothelial Cells

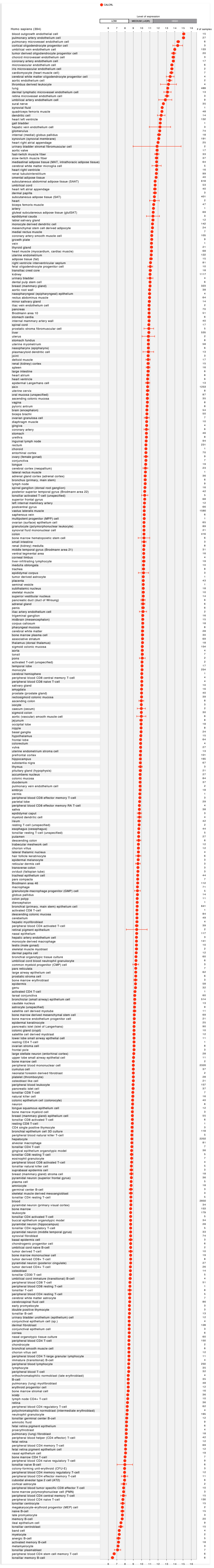

J) Erythroblasts

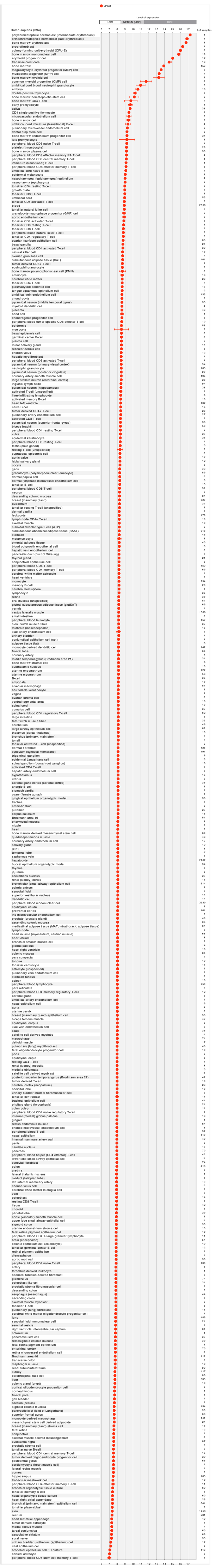

K) Platelets

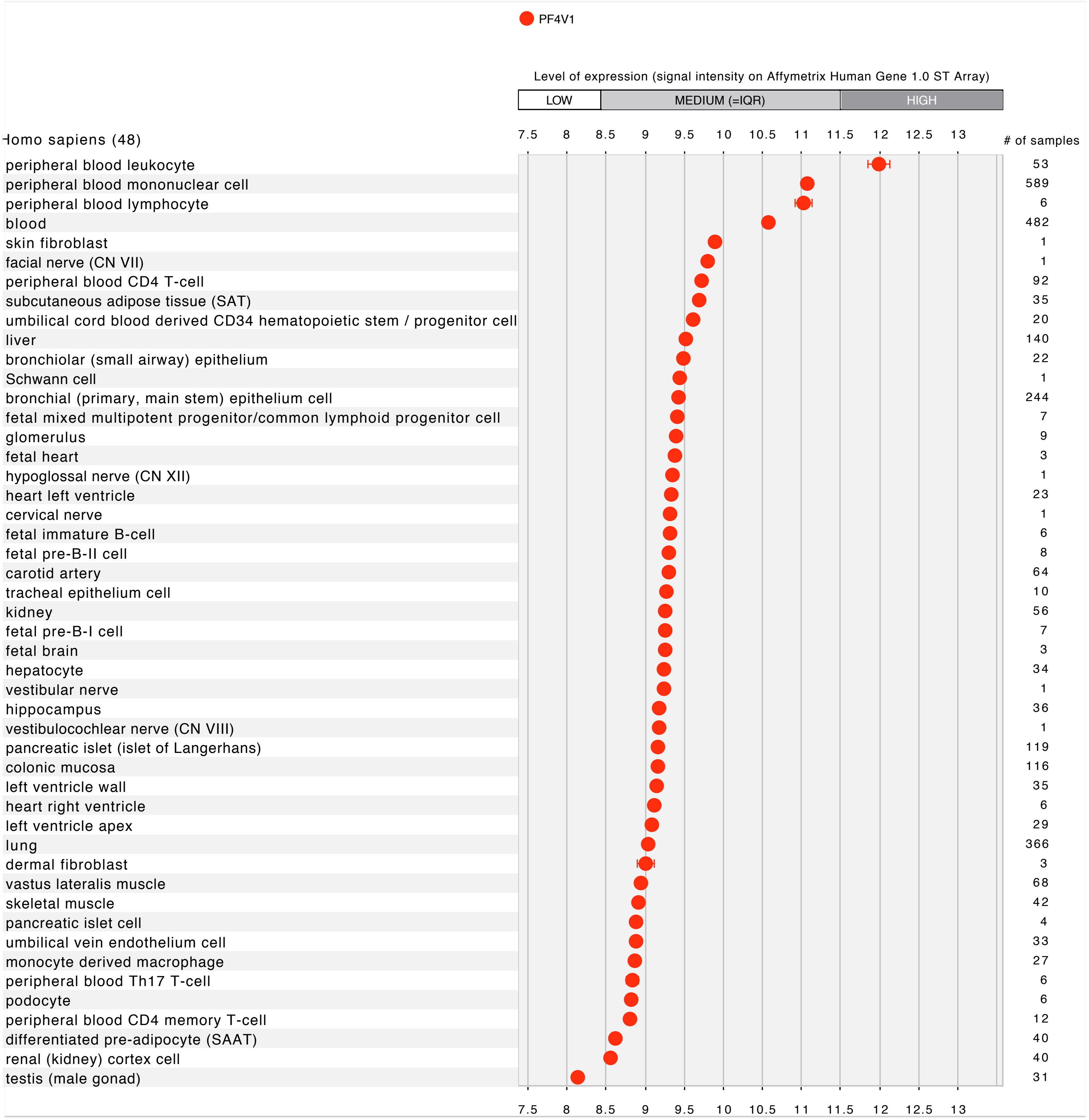

PF4V1
Level of expression (signal intensity on Affymetrix Human Gene 1.0 ST Array)
LOW
MEDIUM (=IQR)
HIGH
Homo sapiens (48)
7.5 8 8.5 9 9.5 10 10.5 11 11.5 12 12.5 13
# of samples
peripheral blood leukocyte 53
peripheral blood mononuclear cell 589
peripheral blood lymphocyte 6
blood 482
skin fibroblast 1
facial nerve (CN VII) 1
peripheral blood CD4 T-cell 92
subcutaneous adipose tissue (SAT) 35
umbilical cord blood derived CD34 hematopoietic stem / progenitor cell 20
liver 140
bronchiolar (small airway) epithelium 22
Schwann cell 1
bronchial (primary, main stem) epithelium cell 244
fetal mixed multipotent progenitor/common lymphoid progenitor cell 7
glomerulus 9
fetal heart 3
hypoglossal nerve (CN XII) 1
heart left ventricle 23
cervical nerve 1
fetal immature B-cell 6
fetal pre-B-II cell 8
carotid artery 64
tracheal epithelium cell 10
kidney 56
fetal pre-B-I cell 7
fetal brain 3
hepatocyte 34
vestibular nerve 1
hippocampus 36
vestibulocochlear nerve (CN VIII) 1
pancreatic islet (islet of Langerhans) 119
colonic mucosa 116
left ventricle wall 35
heart right ventricle 6
left ventricle apex 29
lung 366
dermal fibroblast 3
vastus lateralis muscle 68
skeletal muscle 42
pancreatic islet cell 4
umbilical vein endothelium cell 33
monocyte derived macrophage 27
peripheral blood Th17 T-cell 6
podocyte 6
peripheral blood CD4 memory T-cell 12
differentiated pre-adipocyte (SAAT) 40
renal (kidney) cortex cell 40
testis (male gonad) 31
7.5 8 8.5 9 9.5 10 10.5 11 11.5 12 12.5 13

L) Natural Killer Cells

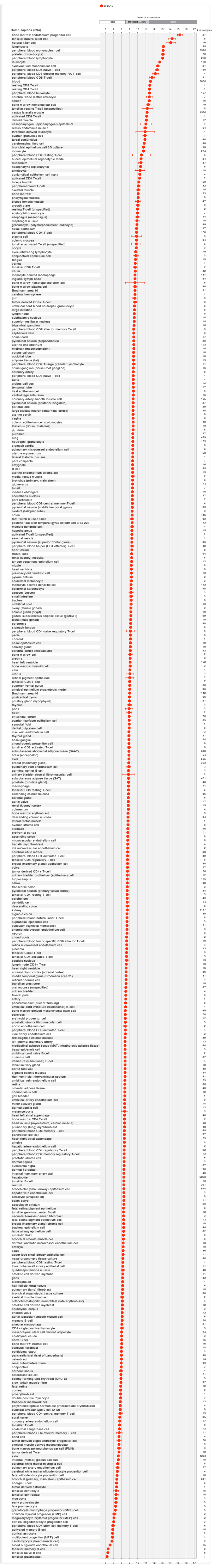

M) CD4+ T cells

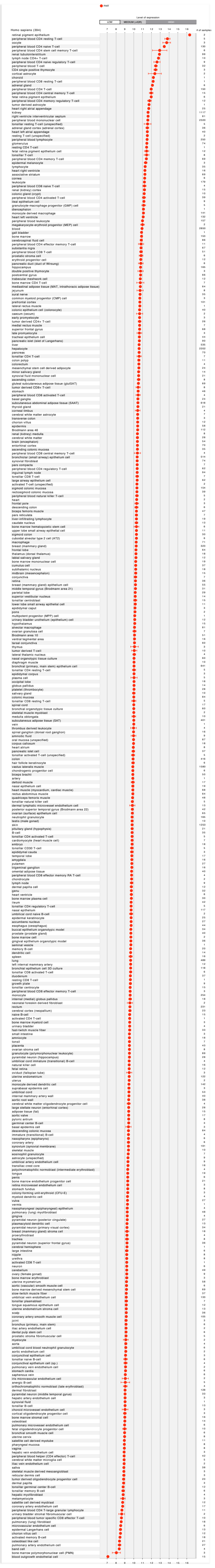

N) CD8+ T cells

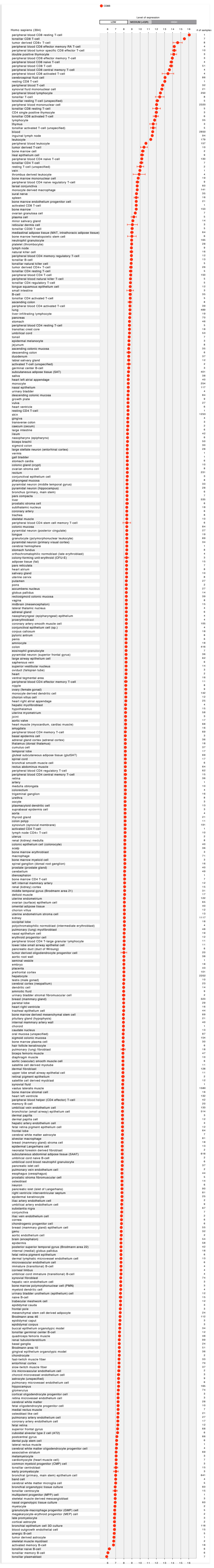

O) B Cells

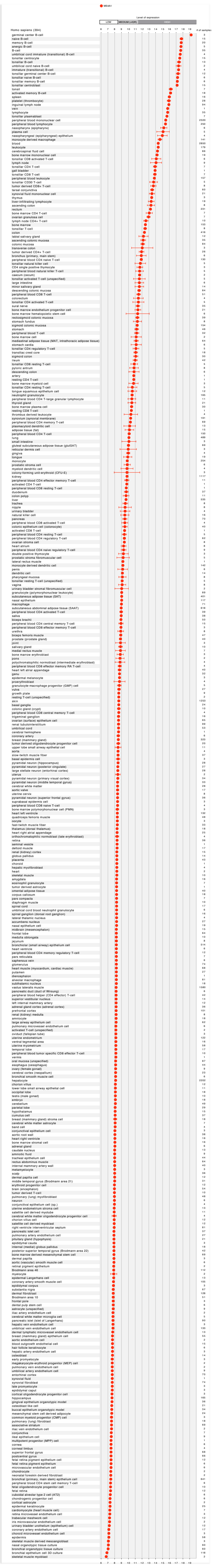

P) Plasmacytoid Dendritic Cells

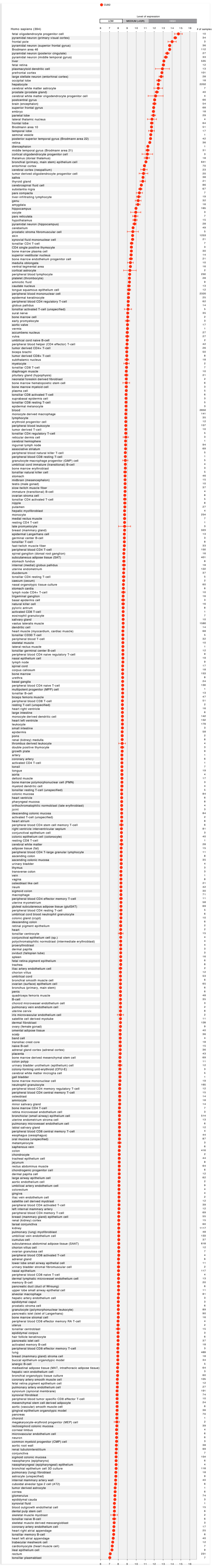

Q) Eosinophils

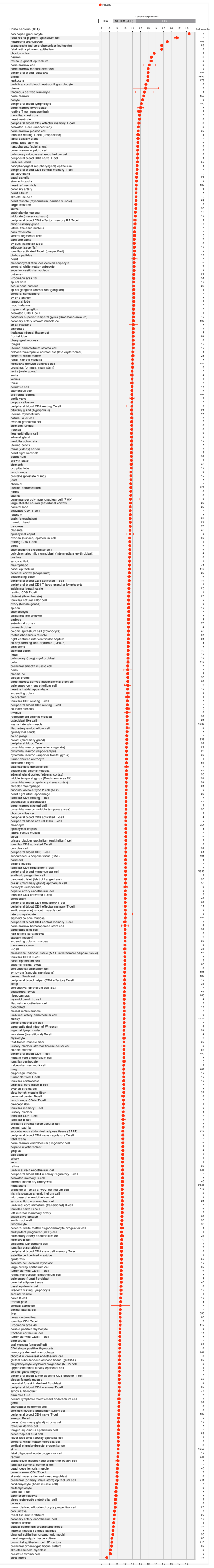

R) Neutrophils

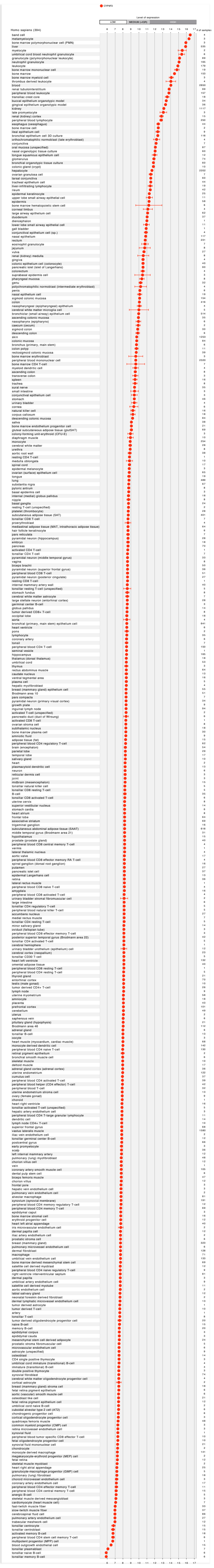

S) Myeloid Dendritic Cells

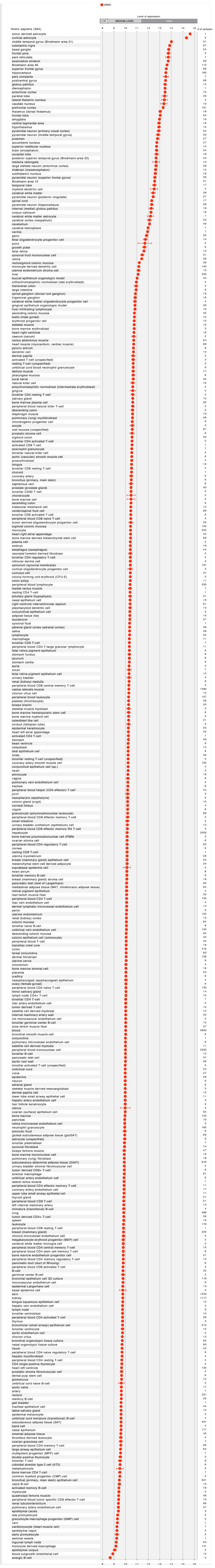

U) Monocytes

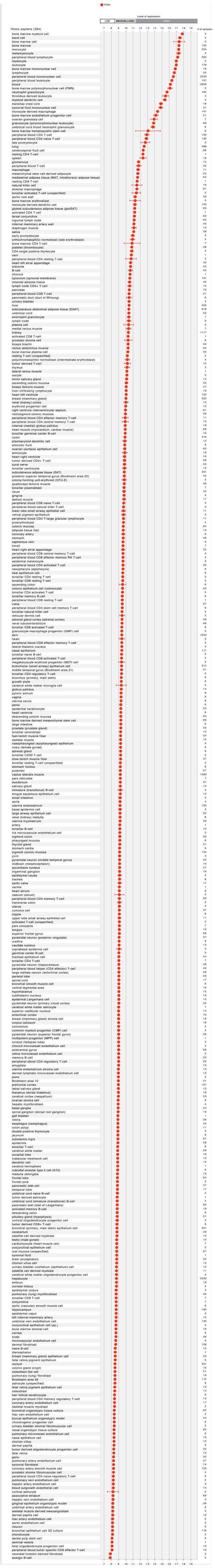

V) Macrophages

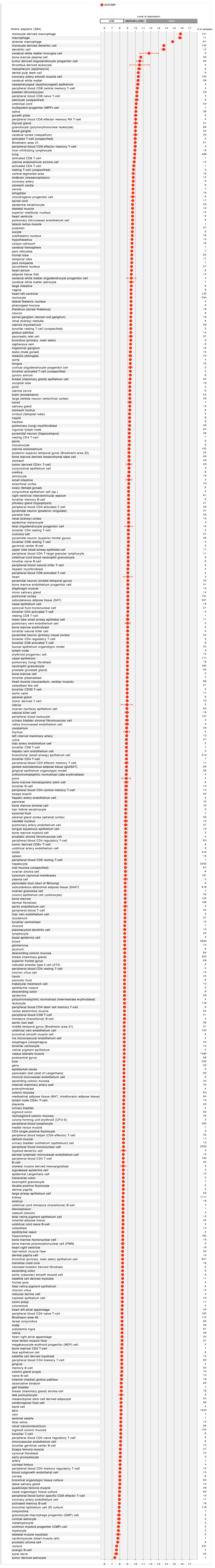

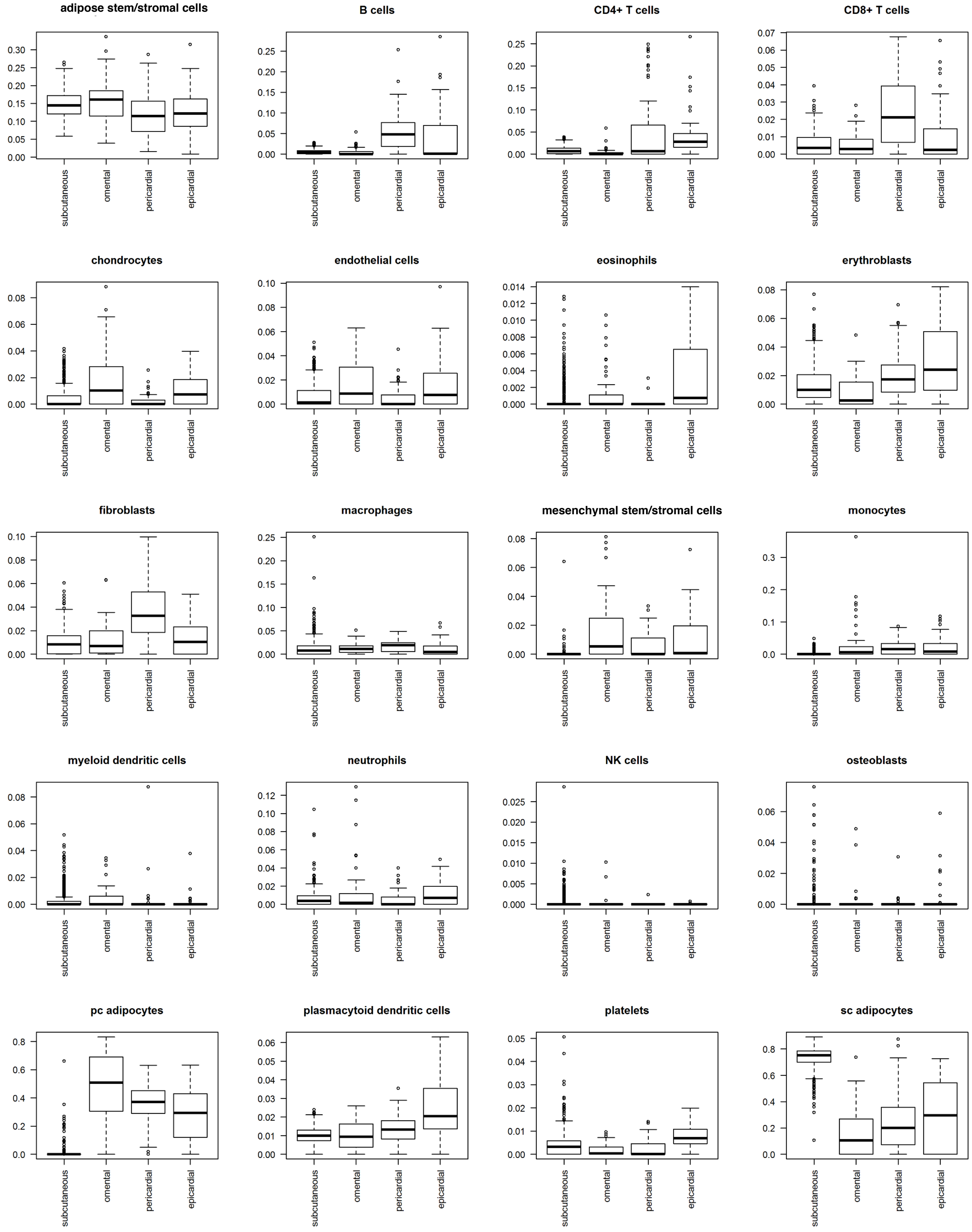

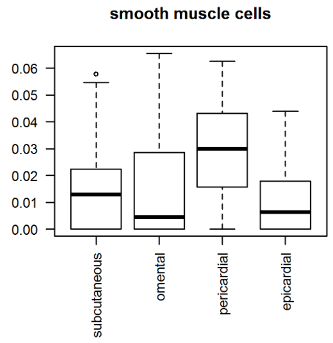

Supplementary Figure S4 : Estimated fractions of cell types per adipose depot showing boxplots for all 21 cell types in the four analyzed depots. Boxplots show medians (thick black lines), upper and lower quartiles (boxes), maximum and minimum values. Corresponding mean values are depicted in Figure 5 in the main text.

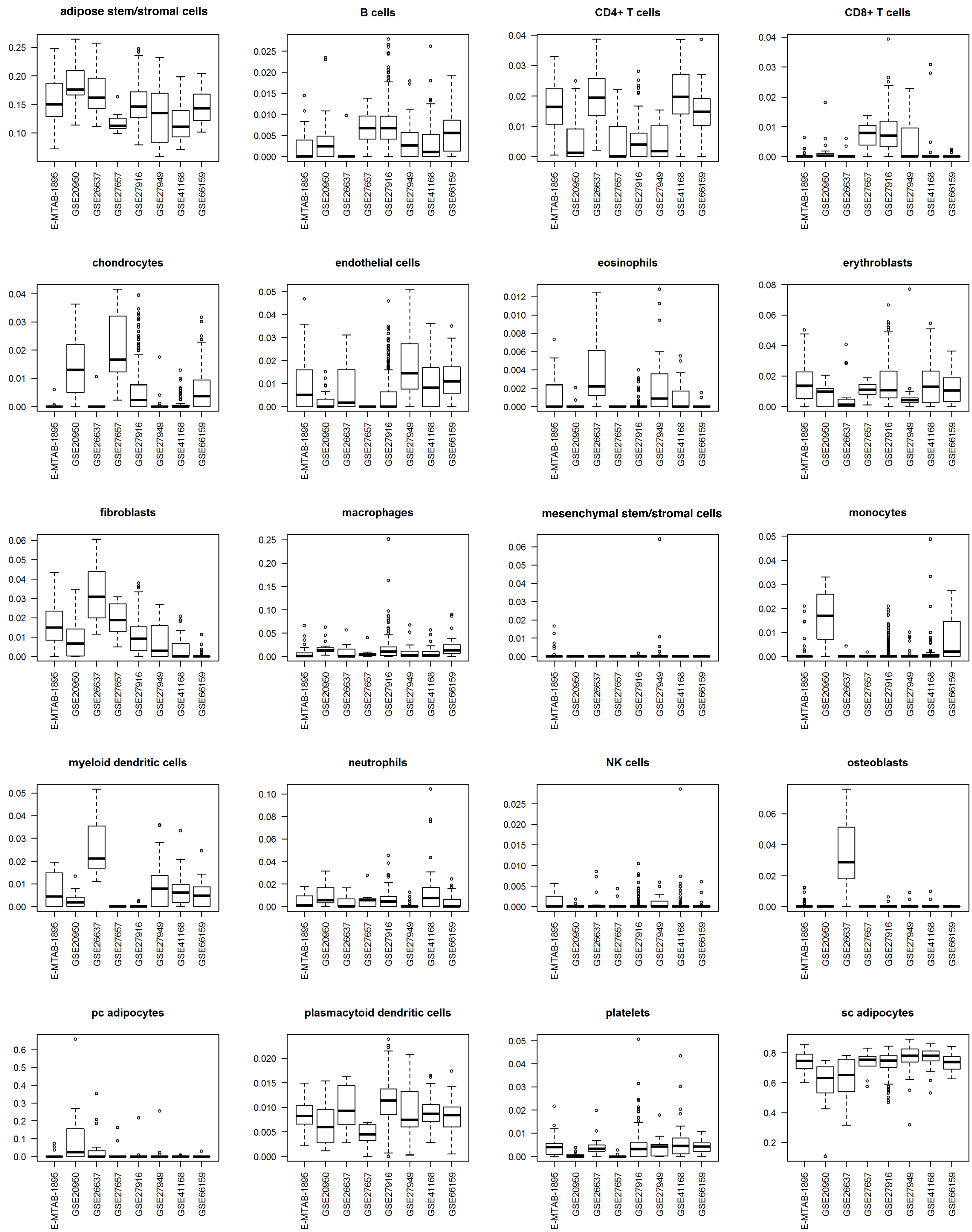

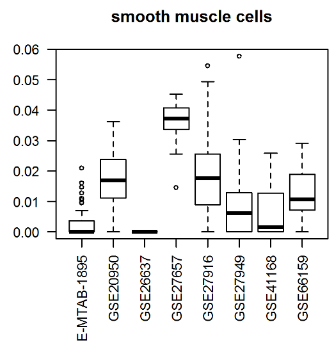

Supplementary Figure S5: Comparison of CIBERSORT estimates of SAT cell type composition from 8 different studies. The inter-study differences are relatively small compared to the reported differences in literature (Figure 4). Inter-study differences partially reflect difference in actual tissue composition and partially batch effects due to different sample handling.

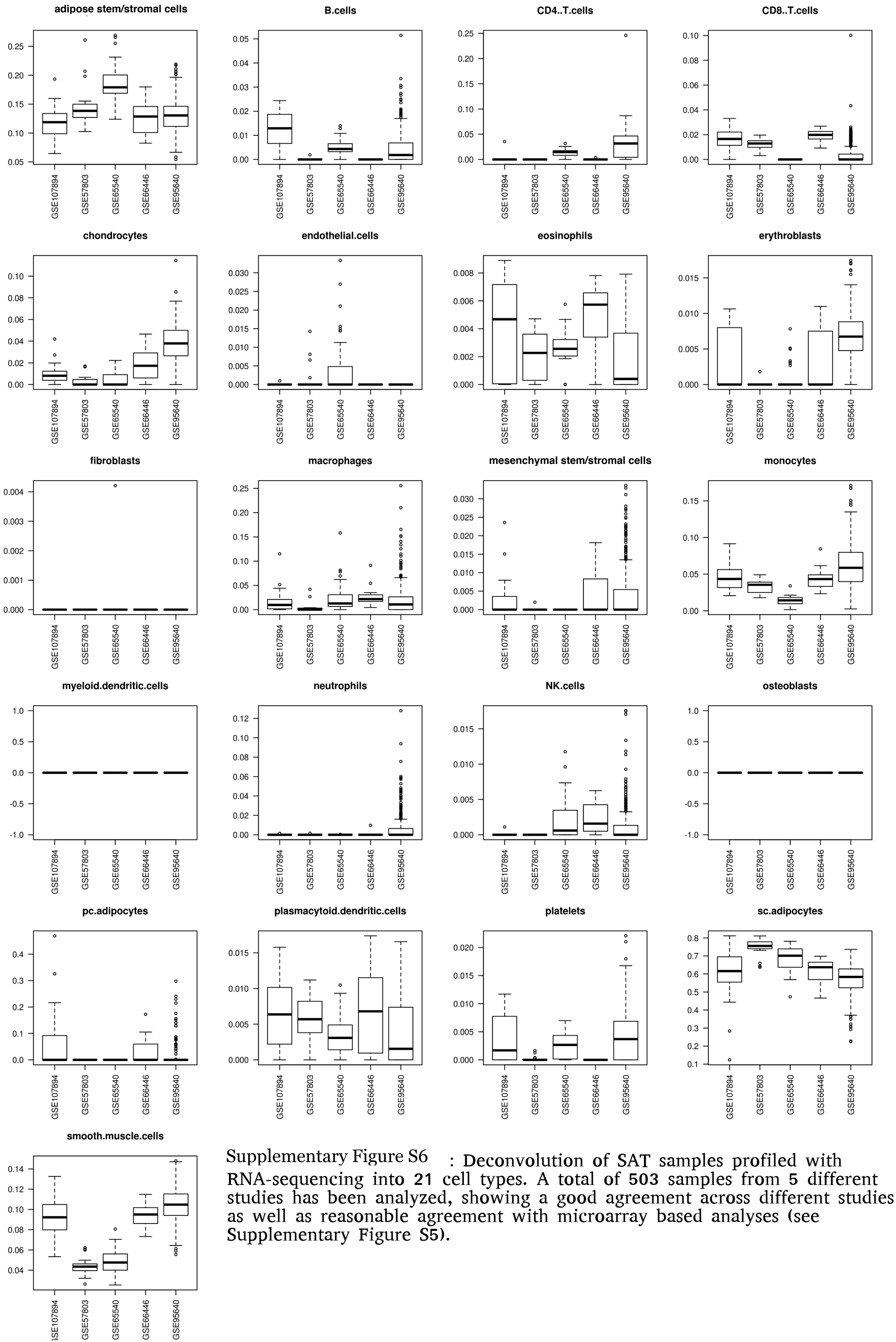

Supplementary Figure S6 : Deconvolution of SAT samples profiled with RNA-sequencing into 21 cell types. A total of 503 samples from 5 different studies has been analyzed, showing a good agreement across different studies as well as reasonable agreement with microarray based analyses (see Supplementary Figure S5).

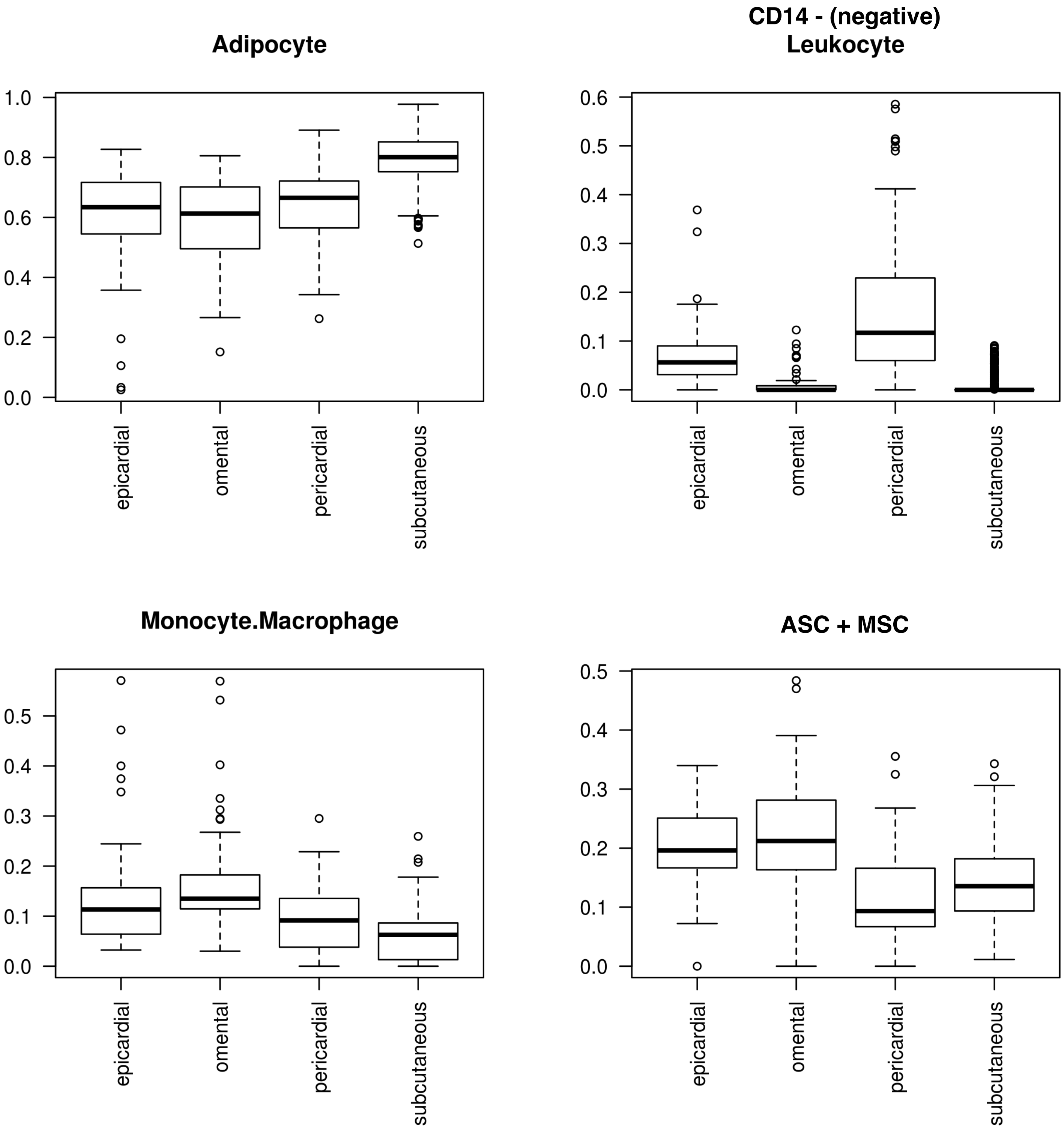

Supplementary Figure S7 : Estimated cell type fractions per adipose depot showing boxplots for the four cell fractions from the ex-vivo reference dataset.

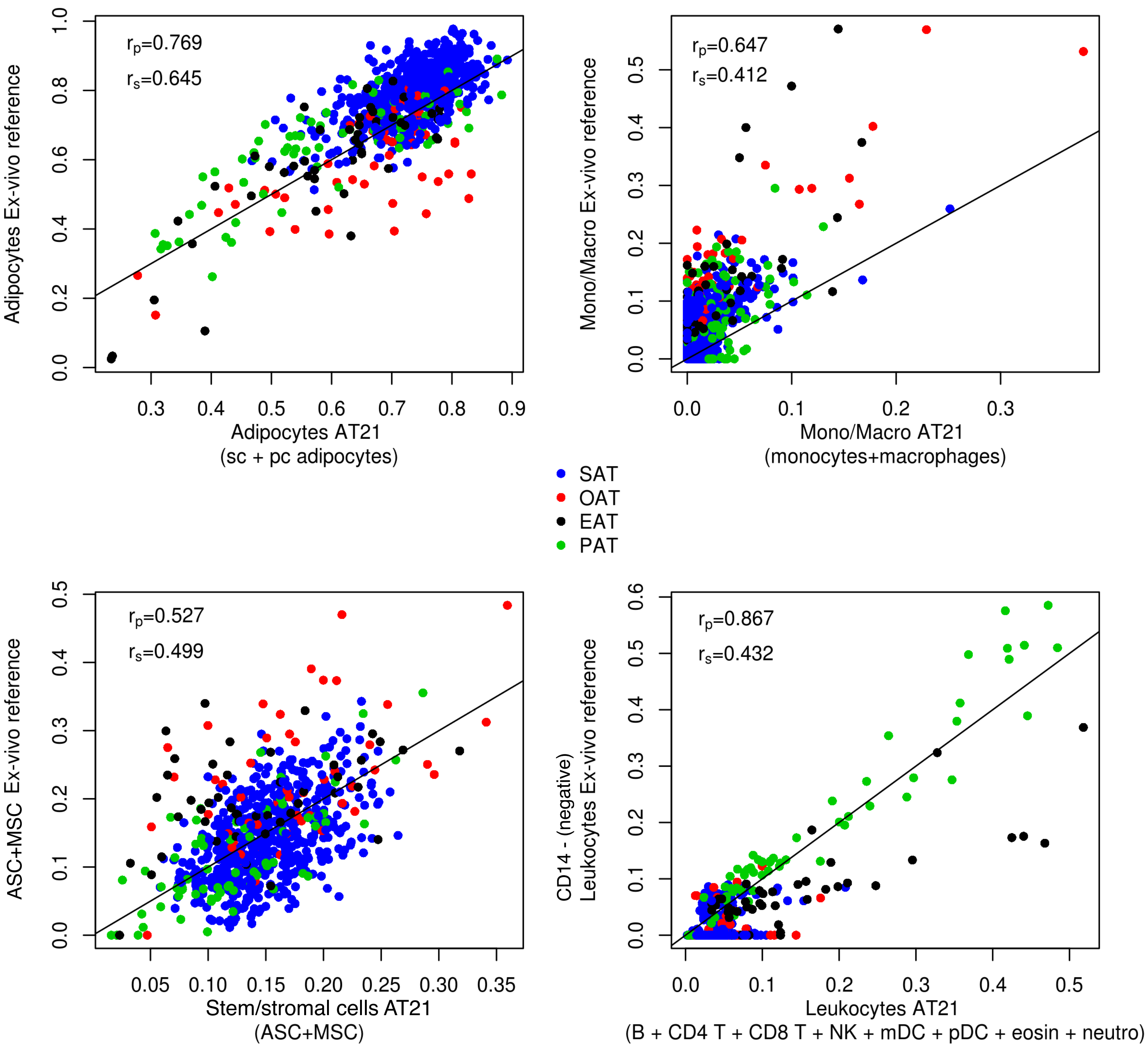

Supplementary Figure S8 : Scatterplot comparing the cell type estimates based on the AT21 signature matrix to the estimates based on the ex-vivo reference dataset for 779 microarray adipose tissue samples from four different adipose tissue depots. For the AT21 estimates, cell fractions have been added up to resemble the less detailed cell fractions represented in the ex-vivo reference dataset.

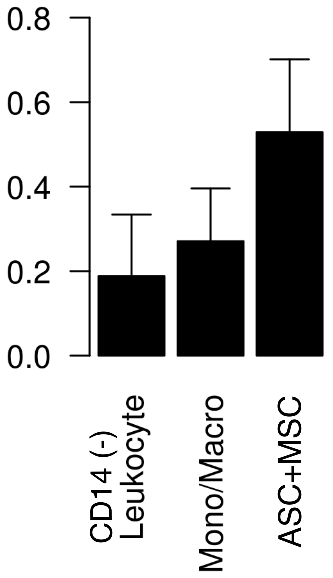

Supplementary Figure S9 : Barplot of estimated cell fractions (mean and standard deviation) from adipose tissue stromal vascular fraction (SVF) deconvoluted using the ex-vivo reference dataset.

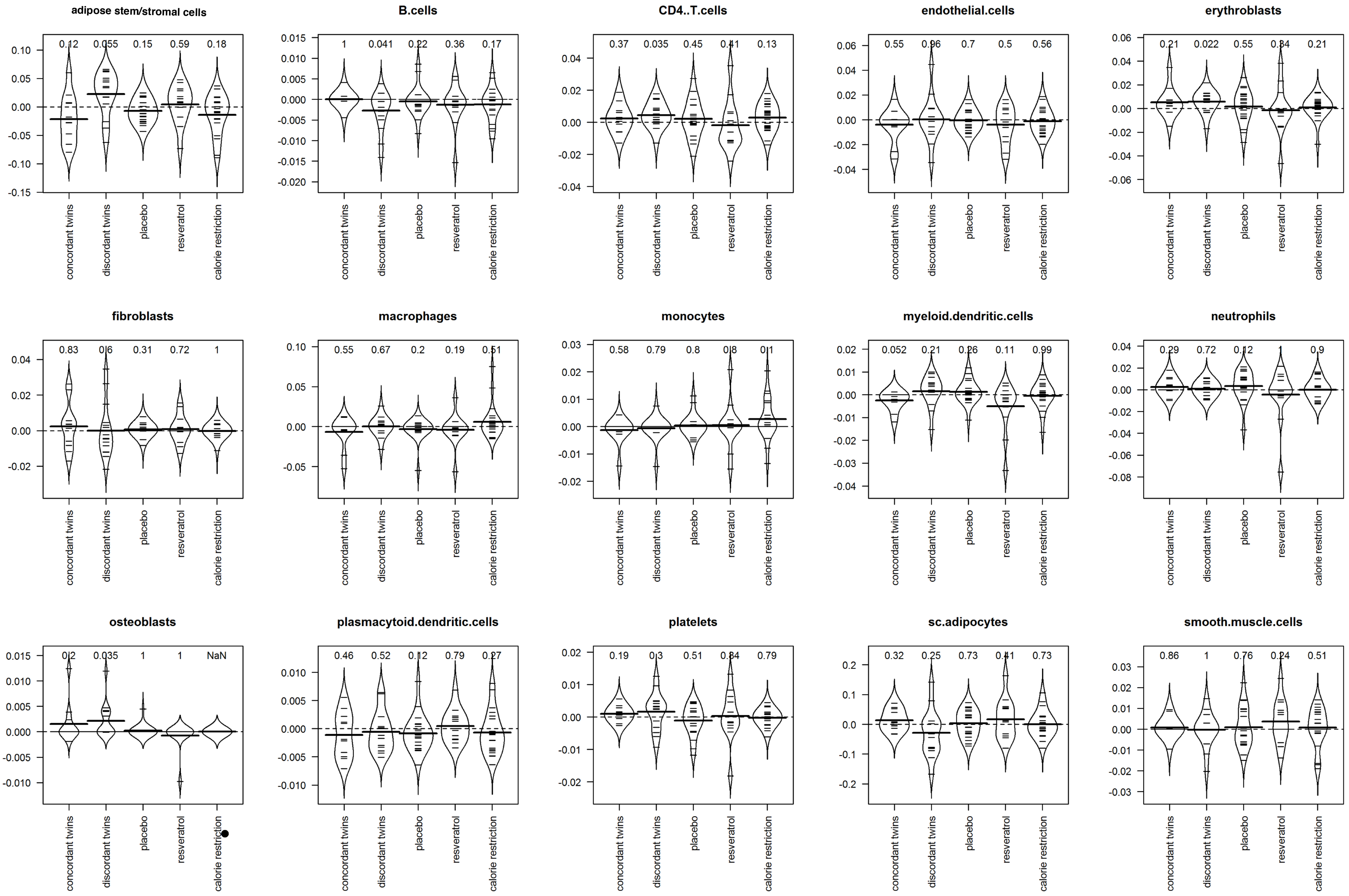

Supplementary Figure S10 : Bean plots showing the population distribution of the cell type percentages for the heavier vs leaner twin, discordant study. The y axis describes differences in estimated cell fractions between the heavier and leaner twin within a pair. P-values are provided above each bean.
