# Supplementary material for: Adipose tissue in health and disease through the lens of its building blocks
Source: Sci Rep. 2020 Jun 26;10:10433. doi: 10.1038/s41598-020-67177-1 (PMC7319996; doi:10.1038/s41598-020-67177-1)
Supplement: Supplementary file 8 — Supplementary Methods [file 41598_2020_67177_MOESM8_ESM.pdf]

Supplementary Methods

Adipose tissue in health and disease through the lens of its building blocks

Authors: Michael Lenz (ML)[1,2,3,‡] , Ilja C.W. Arts (IA)[1,4], Ralf L.M. Peeters (RP)[1,5], Theo M. de Kok (TK)[1,6] and Gökhan Ertaylan (GE)[1,7,‡]

Affiliations:

[1]Maastricht Centre for Systems Biology (MaCSBio), Maastricht University, the Netherlands

[2]Institute of Organismic and Molecular Evolution, Johannes Gutenberg University Mainz, Mainz, Germany

[3]Preventive Cardiology and Preventive Medicine – Center for Cardiology, University Medical Center of the Johannes Gutenberg-University Mainz, Mainz, Germany

[4]Dept. of Epidemiology, CARIM School of Cardiovascular Diseases, Maastricht University, the Netherlands

[5]Dept. of Data Science & Knowledge Engineering, Maastricht University, the Netherlands

[6]Dept. of Toxicogenomics, GROW School of Oncology and Developmental Biology, Maastricht University, Maastricht, The Netherlands

[7]Health, Flemish Institute for Technological Research (VITO), Mol, Belgium

[‡]Corresponding author

Email(s): ML: mlenz@uni-mainz.de;

IA: ilja.arts@maastrichtuniversity.nl;

RP: ralf.peeters@maastrichtuniversity.nl;

TK: t.dekok@maastrichtuniversity.nl;

GE: gokhan.ertaylan@vito.be

Generation of signature matrices

For the generation of AT21, we collected single cell type gene expression data from 21 different cell types (204 samples in total) from publicly available datasets in the Gene Expression Omnibus (GEO) database [S1] (Figure 2A). For AT4, a single dataset with four different cell fractions was utilized. For each signature matrix, raw data (CEL files) of the determined reference dataset were downloaded and preprocessed with Affymetrix Power Tools (https://www.thermofisher.com/nl/en/home/life-science/microarray-analysis/microarray-analysis-partners-programs/affymetrix-developers-network/affymetrix-power-tools.html#) using the robust multi-array average (RMA) normalization method.

The normalized reference dataset was then used to generate the AT21 or AT4 signature matrix using CIBERSORT [S2] (https://cibersort.stanford.edu). For each cell type, CIBERSORT first filters probes based on their differential expression between the selected cell type and all other samples (q value <0.3 (false discovery rate), two-sided unequal variance t-test). Subsequently, probes are ranked according to their fold change between the respective cell type and all other samples and the top G probes are included in the signature matrix. Here, G (between 50 and 150) is selected to minimize the condition number of the signature matrix [S2]. This resulted in a total of 1872 probes in AT21 (Supplementary Data S1) and 375 probes in AT4.

Adipose tissue samples and deconvolution

For deconvolution of the 779 adipose tissue samples from the Affymetrix Human U133 Plus 2.0 microarray platform, raw data (CEL-files) from 12 different studies (accession numbers: E-MTAB-1895, GSE20950, GSE26637, GSE27657, GSE27916, GSE27949, GSE40231, GSE41168, GSE66159, GSE71416, GSE82155, GSE9624) were downloaded from GEO [S1] or ArrayExpress [S3] and preprocessed together with the reference dataset as described above. Subsequently, CIBERSORT was used together with our custom AT21 signature matrix to deconvolute the 779 samples, determining their relative cell type composition.

CIBERSORT provides a deconvolution p-value calculated from 1000 bootstrapped permutations [S2], as well as a correlation value, and root-mean squared error (RMSE) per sample. All analyzed samples had a p-value < 0.0001 and RMSE < 1. We also observed high correlation values with a median of 0.742 (IQR: 0.7238 – 0.7582), indicating that the linear combination of cell types contained in the reference dataset could very well reproduce the tissue expression values. We noted that specifically one dataset (GSE26637) consisting of 20 SAT samples had lower correlation values, which coincides with differences in the cell type estimates e.g. for osteoblasts (Supplementary Figure S5) compared to other data sets. Removal of this dataset did not change the overall results (data not shown). The dataset was not considered for the more in-depth analysis relating tissue composition to phenotypic traits.

Independent (ex-vivo) validation of the TissueDecoder Framework

For generation of the "validation dataset", two datasets (GSE73174 and GSE80654, Affymetrix Human Transcriptome Array 2.0) were downloaded (CEL files) and preprocessed together as described above. Subsequently, we performed probe matching via the biomaRt R package for platform transformation and quantile normalized the validation dataset with the reference and analysis datasets form the Affymetrix Human U133 Plus 2.0 microarray. The generated validation dataset contains expression data from $CD4^+$ T cells, $CD8^+$ T cells, $CD14^+$ Monocytes, $CD19^+$ B cells and $CD56^+$ Natural Killer Cells that were isolated from blood, as well as from adipocytes, progenitors/adipose stem cells ($CD45^-CD34^+CD31^-$), and monocytes/macrophages ($CD45^+CD14^+$) that were isolated from adipose tissue.

The TissueDecoder framework is being used to calculate the percentages of the 21 cell types from the AT21 signature matrix in the validation dataset and to evaluate the expression of conventional markers as well as the primary markers reported from CellMaDe. The results are shown in Supplementary Figure S4 and Supplementary Data S2.

Application to RNASeq data

For testing the applicability of AT21 to deconvolve adipose tissue samples profiled via RNA-seq, we downloaded the preprocessed data ((effective) counts or RPKM/FPKM values) from 5 original datasets

(GEO series numbers: GSE107894, GSE57803, GSE65540, GSE66446, GSE95640), containing a total of 503 adipose tissue samples. All expression values were converted to the unit transcripts per million (TPM) according to the following formulas:

$$TPM_{ij}=\frac{FPKM_{ij}}{\sum FPKM_{ij}}*10^{6}$$, for converting FPKM (or RPKM) of gene i and sample j to TPM values

and

$$TPM_{ij}=\frac{\frac{counts_{ij}}{effLength_{i}}}{\sum \frac{counts_{ij}}{effLenght_{i}}}*10^{6}$$, for converting counts to TPM values, where effLength is the effective

gene length.

Furthermore, we mapped all gene identifiers as well as microarray probe names of AT21 to HGNC (HUGO Gene Nomenclature Committee) ids using the biomaRt R package.

As a next step, we applied the AT21-CIBERSORT deconvolution to these RNAseq samples and compared the resulting cellular fractions to those obtained from microarray data of adipose tissue samples.

Evaluation of primary markers via Anatomically-annotated Tissue Expression Profiles

The definition of primary and secondary criteria defined in CellMaDe, depends on the cell types included in the analysis arguably and therefore, can be considered (adipose) tissue-specific, provided that all relevant cell types from the given tissue are included. In order to evaluate the validity of the most promising primary marker identified per cell type, as well as its general applicability across different tissues, we used Genevestigator [S4] to compare the expression of this marker to a large compendium of different cell and tissue types (394 anatomically annotated tissue expression profiles). Genevestigator is a tool, that allows access to a normalized and curated database of publicly available transciptomics profiles and permits reproducible data analysis. It is freely available for analyzing the anatomical tissue expression for candidate genes.

The result of the most promising markers per cell type is shown in Supplementary Figure S3 where the expression of each gene is shown in a compendium of different tissue types. For the displayed figures, scatterplot and list options are selected with a log2 scale. All genes yielded results from all 394 anatomically annotated expression profiles except for the CellMaDe predicted primary marker Platelet Factor 4 Variant 1 (PF4V1) that is predicted for platelets. The expression of PF4V1 was only present in 48 anatomical tissues from the Genevestigator database where platelets were not present.

Unit conversion for literature review

The literature review of quantitative reports about the adipose tissue cellular composition resulted in 25 studies representing cell fractions in several different types of units, which were converted to percent of total cells using the following formulas. Two studies reported cell counts (macrophages) as number per total number of nuclei, counted via immunohistochemistry on tissue slices. We assumed that the number of anucleated cells in adipose tissue is negligible and directly used the reported number as the percent of total cells.

The unit number per 100 adipocytes was used in six of the included studies that determined macrophage frequency via immunohistochemistry. We converted the reported number (x) into percent of total cells (y) via the formula

$$y = \frac{x}{x + 100 + 50} * 100 \quad . \qquad \text{(equation 3)}$$

Here, x+100+50 represents the estimated number of total cells per 100 adipocytes, assuming that there are 50 cells other than adipocytes and macrophages, such as ASCs, other immune cells, and endothelial cells, per 100 adipocytes. Hence, we assume that adipose tissue roughly consists of 2/3 adipocytes and 1/3 other (SVF) cells, as long as the number of macrophages is not significantly high [S5].

Four studies reported cell numbers in the unit number per g of adipose tissue based on flow cytometry or enzymatic isolation. We converted the reported number (z) into number per 100 adipocytes (x) via the formula

$$x = \frac{z}{z_A} * 100 \quad , \qquad \text{(equation 4)}$$

where z_A is the number of adipocytes per g of adipose tissue. Subsequently, we used the formula described above to convert x into percent of total cells (y). We estimated z_A as

$$z_A = \frac{m_A}{V_A * \rho} = \frac{0.95}{\frac{4}{3} * \pi * 50^3 * 0.9196} * 10^{12} = 1{,}972{,}995 \qquad \text{(equation 5)}$$

Here, $m_A = 0.95\,g$ is the assumed weight of adipocytes per gram of adipose tissue, V_A is the average volume of adipocytes assuming that adipocytes are spherical with a radius of 50µm (diameter of 100µm) [S6], and 0.9196 $\frac{kg}{L}$ is the density of fat.

Finally, there were ten studies that used flow cytometry of the SVF to determine cell numbers as a percent of SVF. We converted this number to a percent of total cells through division by three, assuming that adipose tissue consists of roughly 1/3 SVF cells, as described above.

Supplementary References

S1. Barrett T, Wilhite SE, Ledoux P, Evangelista C, Kim IF, Tomashevsky M, Marshall KA, Phillippy KH, Sherman PM, Holko M, et al: NCBI GEO: archive for functional genomics data sets—update. Nucleic Acids Research 2013, 41:D991-D995.

S2. Newman AM, Liu CL, Green MR, Gentles AJ, Feng W, Xu Y, Hoang CD, Diehn M, Alizadeh AA: Robust enumeration of cell subsets from tissue expression profiles. *Nat Meth* 2015, **12:**453-457.

S3. Kolesnikov N, Hastings E, Keays M, Melnichuk O, Tang YA, Williams E, Dylag M, Kurbatova N, Brandizi M, Burdett T, et al: ArrayExpress update—simplifying data submissions. *Nucleic Acids Research* 2015, **43:**D1113-D1116.

S4. Hruz T, Laule O, Szabo G, Wessendorp F, Bleuler S, Oertle L, Widmayer P, Gruissem W, Zimmermann P: Genevestigator V3: A Reference Expression Database for the Meta-Analysis of Transcriptomes. *Advances in Bioinformatics* 2008, 2008:5.

S5. van Harmelen V, Skurk T, Hauner H: Primary culture and differentiation of human adipocyte precursor cells. *Methods Mol Med* 2005, 107:125-135.

S6. Laforest S, Labrecque J, Michaud A, Cianflone K, Tchernof A: Adipocyte size as a determinant of metabolic disease and adipose tissue dysfunction. *Crit Rev Clin Lab Sci* 2015, 52:301-313.
